# Recent Advances in Bio-Based Fluorescent Hydrogels for Adsorption and Sensing of Toxic Heavy Metal Ions

**DOI:** 10.3390/molecules31060957

**Published:** 2026-03-12

**Authors:** Zhixiong Liu, Man Zhang, Haobing Yang, Chunzhen Zhang, Yu Hou, Junling Wang, Peng Fei, Feng Feng, Yu Feng

**Affiliations:** 1 Shanxi Provincial Key Laboratory of Chemical Biosensing, School of Chemistry and Chemical Engineering, Shanxi Datong University, Datong 037009, China; 17835601250@163.com (M.Z.); yanghb202510@163.com (H.Y.); 19903435302@163.com (C.Z.); hy2002122022@163.com (Y.H.); dtwjllmj@sina.com (J.W.); xiaofeiyier@163.com (P.F.); feng-feng64@263.net (F.F.); 2 School of Materials Science and Engineering, Changzhou University, Changzhou 213164, China

**Keywords:** bio-based, hydrogel, adsorbing, sensing, heavy metal ions

## Abstract

Rapid industrialization and global population growth have led to numerous environmental issues. Among these issues, water polluted with toxic heavy metal ions (HMIs) has become a serious problem. Of the various removal methods, adsorption is considered to be one of the most widely used for purifying wastewater due to its simple operation, high adsorption efficiency, low cost and broad applicability. Bio-based hydrogels are becoming increasingly popular for water purification due to the variety of fabrication and modification methods available. These hydrogels act as adsorption aggregators, increasing the local concentration of HMIs. Bio-based fluorescent hydrogels with fluorescent sensors could be further used to sensitively detect the HMIs, accompanied by an obvious fluorescence quenching. The non-radiative energy transfer between the fluorescent sensor and the adsorbed metal ions is responsible for the sensitive detection. In this review, the recent progress of bio-based fluorescent hydrogels for the adsorption and sensing of toxic HMIs is fully summarized. According to the natural hydrogel sources, the bio-based hydrogels, including cellulose-, chitosan-, alginate- and lignin-based hydrogels, are discussed separately. Finally, the challenges, suggestions and opportunities involved in developing novel bio-based fluorescent hydrogels for the adsorption and sensing of toxic HMIs are presented.

## 1. Introduction

Rapid industrialization and global population growth have led to numerous environmental issues, such as global warming, air pollution, water pollution, and soil contamination. Among these, water pollution stands as one of the most severe environmental challenges facing the world today [1]. Its harm is profound and widespread, not only disrupting ecological balance but also endangering human health [2,3]. Industries such as metallurgy, mining, electroplating, and landfill operations generate vast quantities of wastewater. This wastewater contains enormous amounts and diverse types of heavy metal ions (HMIs) that are discharged into water bodies, intensifying heavy metal pollution in these water systems. Unlike organic pollutants that readily decompose, the hazards of HMIs in wastewater manifest as a concealed, long-term, and cumulative process [4,5]. HMIs follow two primary pathways—“water-aquatic organisms-humans/animals” and “water-soil-crops-humans/animals”—to accumulate within top consumers, causing irreversible harm [4]. For example, Hg(II) is a primary cause of Minamata disease, damaging the central nervous system and kidneys, leading to sensory disturbances, motor coordination disorders, and hearing and speech impairments, and causing severe, irreversible effects on fetal brain development [6,7]. Cr(VI) is highly toxic with strong oxidizing properties, causing skin ulcers and nasal septum perforation. It is a potent carcinogen and mutagen [8,9]. Cr(III) has lower toxicity and is an essential trace element for humans, but excessive intake remains harmful. Therefore, removing these HMIs from industrial wastewater holds significant importance for environmental and human health.

To date, several methods have been developed and applied for removing HMIs from wastewater, including ion exchange, chemical precipitation, redox processes, electrodialysis, membrane separation, and electrolytic extraction [10,11]. However, these methods suffer from drawbacks such as high costs, cumbersome operations, poor stability, and susceptibility to secondary pollution. Adsorption is considered the most practical of the various removal methods due to its easy operation, low cost, high efficiency and broad applicability [12,13]. A variety of adsorbents, such as activated carbon, diatomaceous earth, zeolites, bentonite, metal oxides, synthetic resins, biopolymers, and metal–organic frameworks (MOFs), are used to remove HMIs from wastewater. Many reviews are available on the removal of HMIs using different adsorbents [14,15,16]. However, only a few reviews have discussed hydrogel adsorbents for the adsorption of HMIs [17,18,19]. Therefore, this review mainly focuses on recent progress in the use of hydrogel adsorbents for the removal and sensing of HMIs.

Hydrogels are three-dimensional networks that can retain a large amount of water [20,21]. They are primarily formed through crosslinking polymerization, graft copolymerization, or interpenetrating networks involving polymer backbones and hydrophilic functional groups. Hydrogel adsorbents are simple to prepare and feature abundant pore structures, large specific surface areas, and diverse hydrophilic functional groups [22,23]. Their unique porous structure not only facilitates the rapid diffusion of HMIs but also significantly increases the adsorbent’s specific surface area, thereby providing abundant active adsorption sites. Furthermore, hydrogels often bear abundant hydrophilic functionalities like -COOH, -NH_2_, -OH, -SO_3_H, and -CONH_2_ [24]. These functional groups can strongly bind with the toxic HMIs in wastewater via electrostatic attraction, chemical chelation, or hydrogen bonding and thus achieve efficient HMI removal. In addition, hydrogels themselves are insoluble in water, remain stable in aqueous environments, and can be separated from wastewater to prevent secondary pollution [25]. Consequently, hydrogels are considered one of the most promising adsorbents for removing HMIs from wastewater.

Beyond HMI adsorption, the detection of HMIs in wastewater is also crucial for environmental monitoring and human health protection due to the persistence and toxicity of ions, which can accumulate in ecosystems and cause severe health issues even at trace levels. Nanosized fluorescent sensors like carbon dots (CDs) and metallic nanoclusters possess abundant active sites on their surface that can selectively bind with specific components, resulting in significant fluorescence quenching [26,27,28]. Compared with the traditional chemo-sensors, these nanosized fluorescent sensors have significant advantages, including excellent optical properties, superior hydrophilicity, low toxicity, easy preparation and stability against photobleaching [29,30]. These features enable them to be used as fluorescent sensors for detecting HMIs, dye molecules, and bioactive molecules. Introducing these nanosized fluorescent sensors into a hydrogel network endows the hydrogels with unique fluorescent properties. Importantly, these fluorescent hydrogels can act as adsorption aggregators, increasing the local HMI concentration and resulting in obvious fluorescence quenching, thus enabling sensitive HMI detection [31]. Therefore, these hydrogels can simultaneously remove and detect toxic HMIs in wastewater.

**Scheme 1:**
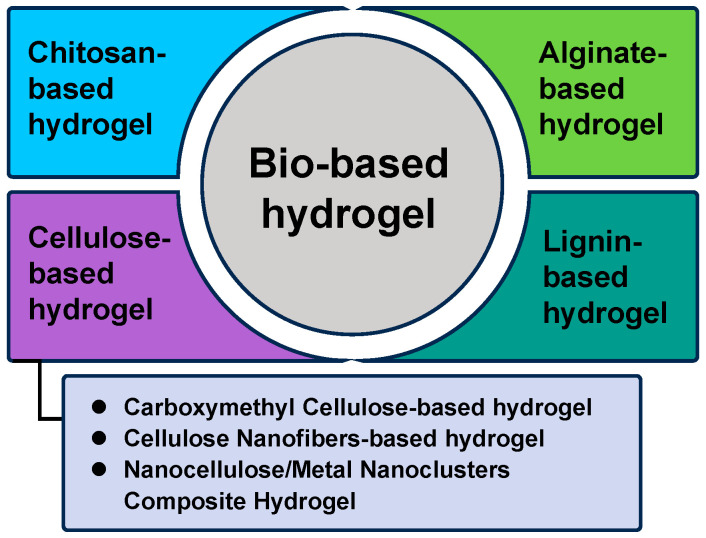
Classification of bio-based fluorescent hydrogels for the adsorption and sensing of HMIs.

Today, most of these functional hydrogel adsorbents are made of synthetic polymers constructed by the polymerization of acrylamide (AM), acrylic acid (AA), acrylic esters (AE), and vinyl alcohol (VA) as monomers, or through the artificial synthesis of macromolecules (e.g., polyvinyl alcohol, polyethylene glycol, etc.) [17]. However, these synthetic hydrogels suffer from many drawbacks, such as being non-degradable, biologically toxic, and unable to absorb amphoteric HMIs. Therefore, the bio-based hydrogel adsorbents derived from natural biomass have attracted intensive interest for their ability to remove HMIs from wastewater due to their being widely available, environmentally friendly, cost effective, free from secondary pollution, biodegradable and non-toxic [19,32,33,34]. Natural hydrogel adsorbents are rich in various moieties, including hydroxyl, carboxyl, and amino functional moieties as active adsorbing sites, and the 3D network structure provides numerous diffusion pathways facilitating HMI removal. Recently, numerous bio-based hydrogels derived from various biomaterials, including cellulose, agar, chitosan, gelatin, starch, alginates, hyaluronic acid, and silk protein, have been widely used as adsorbents for removing and sensing HMIs in wastewater. In this review, we mainly focused on research progress in the use of bio-based hydrogels for the adsorption and sensing of toxic HMIs over the past decade, from 2015 to 2025. We grouped bio-based hydrogels into four categories based on their natural biomass sources (Scheme 1): (1) cellulose-, (2) chitosan-, (3) alginate-, and (4) lignin-based hydrogels. We fully discussed the design strategy of the composite hydrogels, their adsorption performance, their ability to detect HMIs and their adsorption and detection mechanisms. Finally, the current challenges, limitations and potential outlooks in bio-based hydrogel adsorbents were further discussed.

## 2. Classification of Bio-Based Hydrogels

To achieve highly efficient adsorption, hydrogel adsorbents with superior properties like large specific surface area, good mechanical strength, abundant adsorption sites, readily available sources, regenerability, and biodegradability are the ideal choice. Bio-based hydrogels, which exhibit almost all of the above features, are considered an exceptional option for the removal of HMIs. At present, toxic HMIs are being successfully removed by natural polymer-based double-network hydrogels with high mechanical strength. Notably, these natural hydrogel adsorbents with fluorescent probe units can also be used to detect HMIs by obvious fluorescence quenching due to the interactions between the adsorbed HMIs and fluorescent probe units. Therefore, these fluorescent bio-based hydrogels can remove and detect toxic HMIs in wastewater simultaneously.

### 2.1. Cellulose-Based Hydrogels

Cellulose is a natural polysaccharide consisting of many D-glucose units joined together by 1,4-β-glycosidic bonds. As the world’s most abundant natural polymer, cellulose constitutes the main component of plant cell walls and is predominantly found in plants and algae [35]. It boasts an abundance of sources and is low-cost, renewable, biodegradable, highly biocompatible and environmentally friendly. The numerous -OH groups in the cellulose structure make it reactive toward pollutants such as HMIs. However, natural cellulose possesses a highly ordered crystalline structure and a high polymerization degree, which reduces its specific surface area and adsorption sites [36]. Consequently, raw cellulose exhibits poor adsorption capacity. To increase its adsorption capability, many different approaches to modify cellulose have been reported, such as esterification, etherification, oxidation, and graft copolymerization [37].

#### 2.1.1. Carboxymethyl Cellulose-Based Hydrogels

Carboxymethyl cellulose (CMC) is a water-soluble anionic polymer obtained through an etherification reaction using cellulose and chloroacetic acid (or its sodium salt) as starting materials under alkaline conditions [38]. However, the raw CMC-based hydrogels exhibit poor adsorption of HMIs due to limited adsorptive sites and weak mechanical strength. An efficient approach to improving the adsorption capacity is to introduce a second polymer chain to form an interpenetrating double network [39].

**Figure 1:**
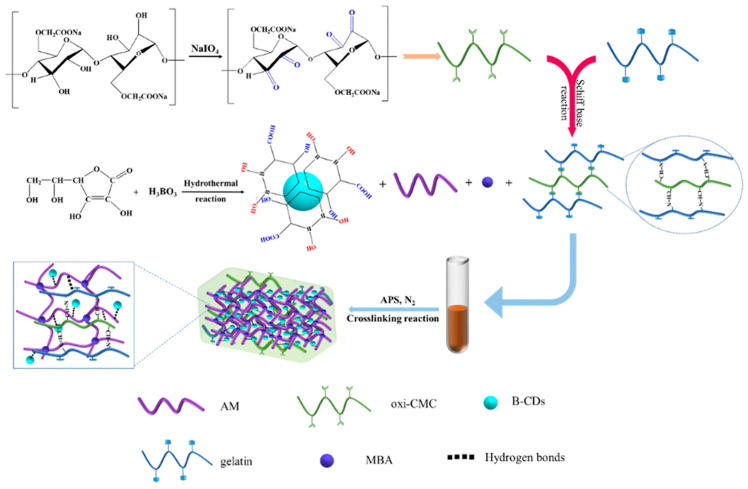
A schematic of the preparation process of the self-healing fluorescent double network hydrogel. Reprinted with permission from Ref. [40]. Copyright 2008 Elsevier.

He et al. developed a self-healing fluorescent double network hydrogel (BCS) from oxidized CMC [40] (Figure 1). Boron-doped carbon dots (B-CDs) were first introduced into the polyacrylamide network. A second network consisting of oxidized CMC and gelatin, through the Schiff base reaction, was interpenetrated with the first hydrogel network. The BCS hydrogel showed efficient adsorption of Ag(I) with a maximum adsorption capacity of 407 mg/g. The fluorescent BCS hydrogel exhibited sensitive detection of Ag(I), with a linear fluorescence response that ranged from 0 to 75 μM and a detection limit (LOD) of 3.798 μM. The BCS hydrogel exhibited excellent self-healing properties, with no loss of adsorption capacity after seven cycles.

**Figure 2:**
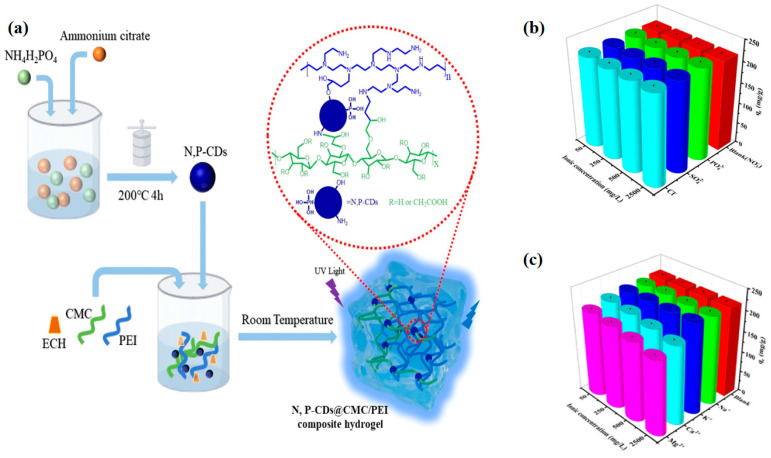
(**a**) A schematic of the preparation of N, P-CDs@CMC/PEI composite hydrogels. (**b**) The effect of various anions on Cr(VI) adsorption. (**c**) The effect of different cations on Cr(VI) adsorption. Reprinted with permission from Ref. [25]. Copyright 2008 Elsevier.

Chu et al. developed a fluorescent composite hydrogel (N, P-CDs@CMC/PEI) to adsorb and detect toxic Hg(II) and Cr(VI) [25] (Figure 2). The composite hydrogel exhibited an interpenetrating network structure, with CMC acting as the primary matrix and PEI forming a secondary network (Figure 2a). The maximum adsorption capacities for Cr(VI) and Hg(II) were 289.5 and 846.7 mg g^−1^, respectively. The relationship between the type of adsorbent, pH value, HMI concentration, and temperature and the adsorption capacity of the composite hydrogels was studied. The pseudo-first-order and pseudo-second-order kinetic models were chosen to fit the adsorption isotherms. The adsorption of Cr(VI) and Hg(II) by the hydrogels was fitted well to the pseudo-second-order model, suggesting that chemical adsorption was responsible for the adsorption process. To further determine the rate-determining step in the adsorption process, the intraparticle diffusion model was chosen to fit the experimental data. The results indicated that intraparticle diffusion was not the sole rate-determining step but was also affected by membrane diffusion in the adsorption process. Isothermal adsorption suggested that the Langmuir model was applicable to explain the adsorption process for Hg(II) and Cr(VI), suggesting a homogeneous monolayer adsorption. Thermodynamic study indicated that a negative Gibbs free energy change suggested that Cr(VI) adsorption was a spontaneous process. The positive entropy change indicated that randomness at the solid/liquid interface increased during Cr(VI) adsorption. The presence of competitive organic and inorganic components had no obvious effect on the adsorption of HMIs at low concentrations (Figure 2b,c). The adsorption ability of the hydrogel sample for Hg(II) and Cr(VI) remained high after four cycles, indicating a good regeneration ability. FT-IR and XPS analyses of the composite hydrogel after capture of the above two toxic HMIs were performed to clarify the adsorption mechanism. In acidic conditions, the Cr(VI) anions were found to exist in the form of HCrO_4_^−^. The HCrO_4_^−^ species were strongly adsorbed on the composite hydrogel due to electrostatic attraction. Some adsorbed HCrO_4_^−^ was reduced to Cr(III) due to the reduction effect of the hydroxyl group, which then chelated with heteroatoms like N and O on the hydrogel surface and finally was immobilized in the hydrogel network. The excellent adsorption of the composite hydrogel toward Hg(II) was attributed to electrostatic attractions and chelating effects between the heteroatom-containing moieties and Hg(II). Notably, fluorescence quenching occurred as a result of non-radiative energy transfer between the N, P-doped-CDs and the adsorbed HMIs. The limit of detection (LOD) for toxic Hg(II) was 0.48 mg/L within a range from 1 to 200 mg/L.

Recently, Zhao et al. developed a CMC-based fluorescent hydrogel (DCG) by introducing silicon-doped carbon dots into a CMC hydrogel network to detect and adsorb Fe(III) [41]. DCG hydrogel contains a number of adsorption active groups such as -OH, -NH_2_ and -COOH. These groups provided numerous adsorption sites for Fe(III). The calculated maximum adsorption capacity toward Fe(III) was up to 125.30 mg/g. DCG hydrogel showed a superior responsivity toward Fe(III) within the range of 0 to 100 mg/L, with an LOD value of 0.6595 mg/L.

#### 2.1.2. Cellulose Nanofiber-Based Hydrogels

**Figure 3:**
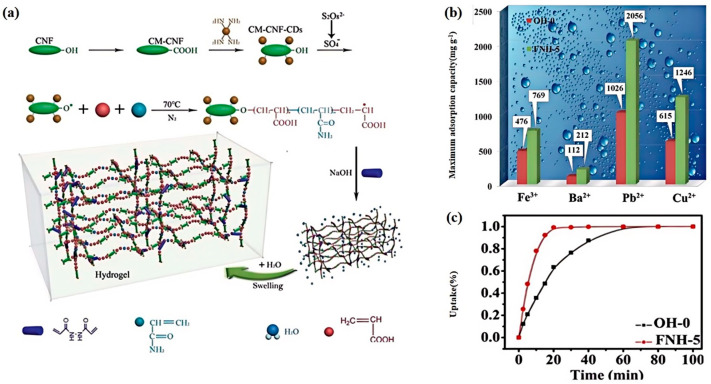
(**a**) Fabrication of the fluorescent CNF-based hydrogel (CDs@CNFs). (**b**) The Fe^3+^, Ba^2+^, Pb^2+^ and Cu^2+^ saturated adsorption by the CDs@CNFs (green columns) and the original hydrogel (red columns). (**c**) Dynamic isotherms of Fe^3+^ adsorption by CDs@CNFs and the original hydrogel. Reprinted with permission from Ref. [31]. Copyright 2008 Elsevier.

Of the various cellulose materials, cellulose nanofibers (CNFs) boast a variety of exceptional properties, such as nanoscale dimensions, high specific strength and stiffness, a large surface area, natural biodegradability, and non-toxicity [42]. CNFs can provide the ‘skeleton’ of the hydrogel network, enabling the formation of a 3D porous structure. They also supply numerous functionalities that act as chelating sites for HMIs. Therefore, CNF is considered an ideal nanomaterial for developing novel bio-based hydrogels with excellent HMI adsorption ability. CNFs are also used as an adsorption-aggregator for HMIs, facilitating the sensitive detection of special HMIs. Guo et al. fabricated a fluorescent CNFs-based hydrogel (CDs@CNFs) for adsorbing and detecting HMIs [31] (Figure 3a). Based on the experimental results, the maximum adsorption capacities for Fe^3+^, Ba^2+^, Pb^2+^ and Cu^2+^ were 769, 212, 2056 and 1246 mg g^−1^, respectively (Figure 3b,c). It also showed a high sensitivity in detecting Fe(III), with an LOD value of 18 mg/L.

**Figure 4:**
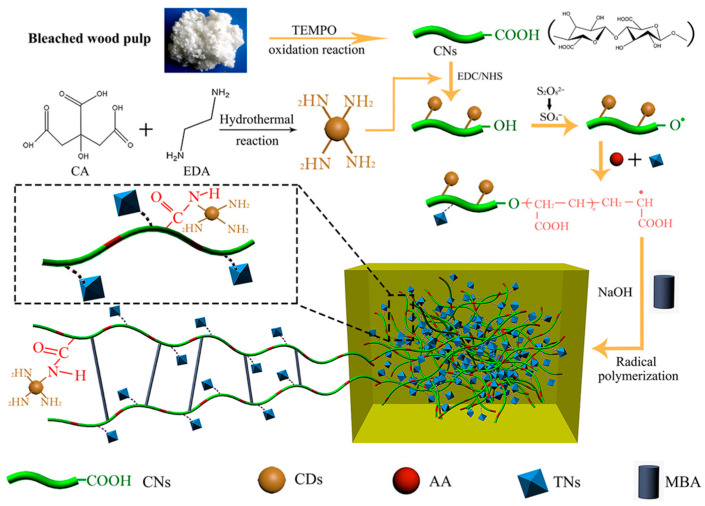
The synthesis scheme for the fluorescent nanocellulosic hydrogel (FNH). Reprinted with permission from Ref. [44]. Copyright 2008 Elsevier.

Titanate nanofibers (TNs) are one-dimensional nanostructures characterized by their chain-like or fibrous morphology. TNs inherently possess a layered tunnel structure and a high specific surface area, exhibiting strong adsorption capacity and ion exchange capability toward HMIs [43]. Incorporating TNs into CNFs-based hydrogels not only improves the mechanical strength but also enlarges the number of adsorption sites. A fluorescent nanocellulosic hydrogel (FNH) with TNs was fabricated for adsorbing and detecting Cr(VI) ions [44] (Figure 4). The FNH hydrogel exhibited a high selectivity toward Cr(VI), with negligible interference from other common metal ions. The maximum adsorption capacity of the FNH toward Cr(VI) reached 648.4 mg/g. The LOD value for Cr(VI) was as low as 0.039 μg/L. After four regenerated cycles, the adsorption capacity remained at 83% of its initial value.

**Figure 5:**
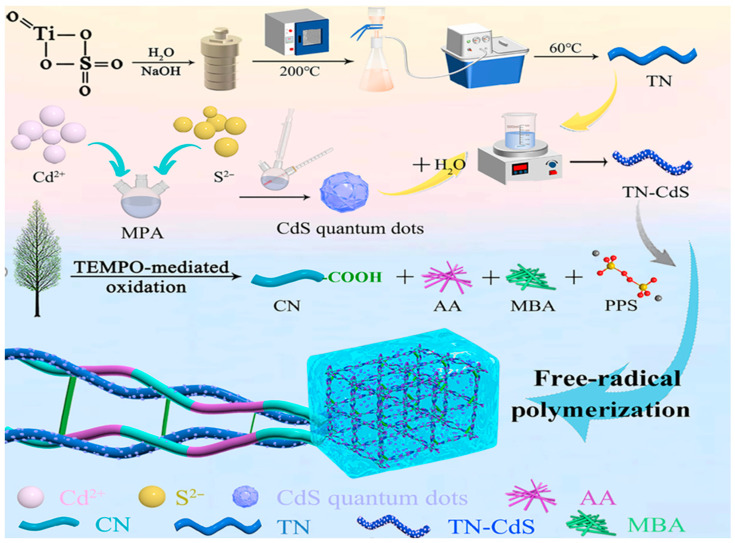
Synthesis scheme of CNFs/titanate nanofiber-modified CdS quantum dot hydrogel (CTH). Reprinted with permission from Ref. [45]. Copyright 2008 Elsevier.

Recently, they synthesized a CNFs/titanate nanofiber-modified CdS quantum dot hydrogel (CTH) [45] (Figure 5). TN modified with CdS quantum dots (TN-CdS) acted as a nanosized photocatalyst that increased the photocatalytic efficiency of the hydrogel. CTH hydrogel exhibited a maximum adsorption capacity of 373.3 ± 14.2 mg/g toward Cr(VI). Concurrently, the photocatalytic reduction rate constant of the CTH was found to be 0.0586 ± 0.0038 min^−1^, greater than that of the TN-CdS. CTH hydrogel exhibited excellent stability and reusability, with an initial removal efficiency of 84.9% even after five cycles. This outstanding removal performance of CTH was attributed to the 3D porous structure formed by CNFs and TN-CdS, enhancing the adsorption and photocatalytic properties.

**Figure 6:**
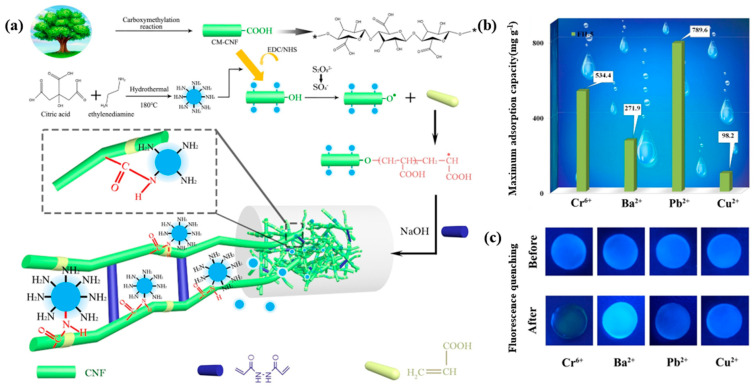
(**a**) The synthetic procedure for the porous fluorescent CNFs-based hydrogel (ACDs@CNFs). (**b**) Maximum sorption capacities of ACDs@CNFs. (**c**) Fluorescence quenching for ACDs@CNFs hydrogel after different HMIs adsorption (Cr^6+^, Ba^2+^, Pb^2+^ and Cu^2+^). Reprinted with permission from Ref. [46]. Copyright 2008 Elsevier.

Luo et al. synthesized a fluorescent CNFs-based hydrogel (ACDs@CNFs) with amino-modified carbon dots (ACDs) to detect and adsorb toxic Cr(VI) [46] (Figure 6a–c). The adsorption performance revealed a maximum adsorption capacity for Cr(VI) up to 534.4 mg/g (Figure 6b). The absolute quantum yield (AQY) of this fluorescent hydrogel showed a good linear relationship with Cr(VI) concentrations ranging from 10 to 50 mg/L, with an LOD value of 4.2 mg/L. In the adsorption process, Cr(VI) aggregated onto the hydrogel surface due to electrostatic attraction. The 3D porous structure of the hydrogel provided transport pathways for Cr(VI) ions, facilitating their diffusion from the external to the internal regions of the hydrogel and adsorption onto internal sites. Some Cr(VI) was transformed into Cr(III) and chelated with the hydroxyl and carboxyl groups on the hydrogel or exchanged with Na(I) ions within the hydrogel. Furthermore, ACDs were encapsulated within the hydrogel to serve as a fluorescent sensor. Cr(VI) was adsorbed onto the hydrogel via electrostatic interactions, leading to fluorescence quenching. A fluorescent CNFs-based hydrogel (FNH) was fabricated for the removal and detection of Fe(III) and Pb(II) (Figure 6c) [47]. The static adsorption experiment showed that FNH removed over 69.4% of Fe(III) and over 98.2% of Pb(II), with adsorption capacities of 98.3 and 442.0 mg/g, respectively. FNH can also be used to detect Fe(III), with an LOD value of 62.5 mg/L.

**Figure 7:**
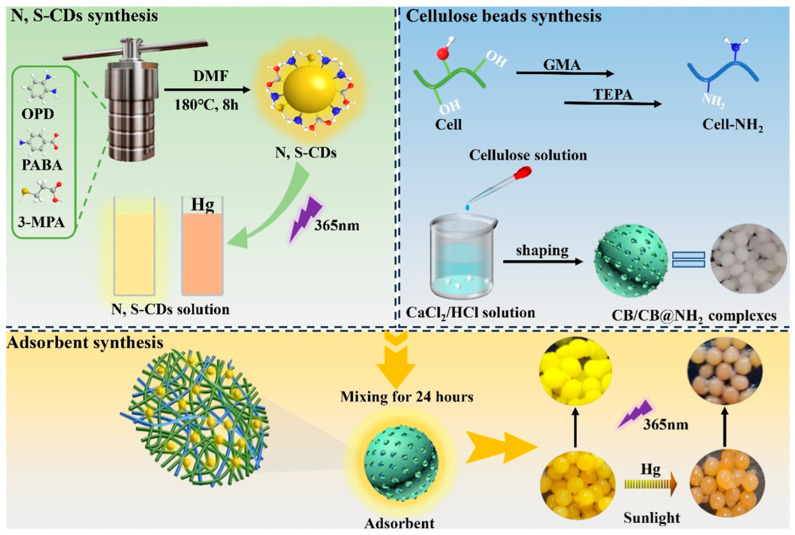
The preparation of fluorescent gel beads adsorbents CNFs-based hybrid hydrogel beads (HHB). Reprinted with permission from Ref. [48]. Copyright 2008 Elsevier.

Li et al. developed a family of CNFs-based hybrid hydrogel beads (HHB) to achieve detection and adsorption of Hg(II) [48] (Figure 7). The hydrogel beads exhibited a maximum adsorption capacity of 290.70 mg/g toward Hg(II). Additionally, the LOD value for Hg(II) was as low as 8.8 × 10^−8^ M. Furthermore, the hybrid hydrogel beads demonstrated excellent stability, and the hydrogel beads maintained their sensitive and selective detection for Hg(II) even after two months of storage. In addition, the adsorption of Hg(II) remained over 80% after five consecutive adsorption–desorption cycles, demonstrating its durability and suitability for long-term environmental applications.

#### 2.1.3. Nanocellulose/Metal Nanoclusters Composite Hydrogel

Metal nanoclusters are a new class of fluorescent nanomaterial composed of several to tens of metallic atoms. These nanoclusters are extremely small (typically less than 2 nm in size) and show excellent fluorescent properties, biocompatibility, and low toxicity [49]. However, they also suffer from easy aggregation and difficult storage. Incorporating metal nanoclusters into the hydrogel matrix can not only effectively address this issue but also serve as a fluorescent probe to detect toxic HMIs. In addition, functionalities on the nanocluster can interact with the bio-based hydrophilic polymer chains through hydrogen bonding and hydrophobic interactions, acting as an additional dynamic physical crosslinking point to improve their mechanical strength.

**Figure 8:**
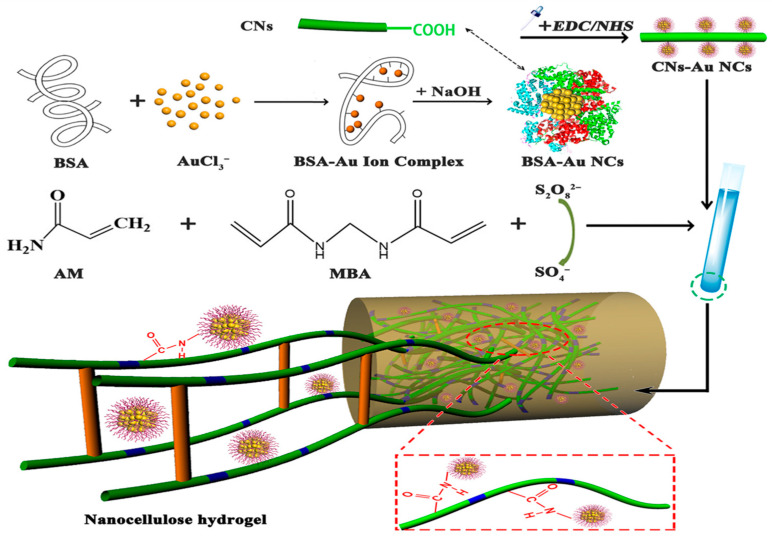
A schematic diagram of the preparation of a fluorescent nanocellulose hydrogel (NHB). Reprinted with permission from Ref. [50]. Copyright 2008 Elsevier.

**Figure 9:**
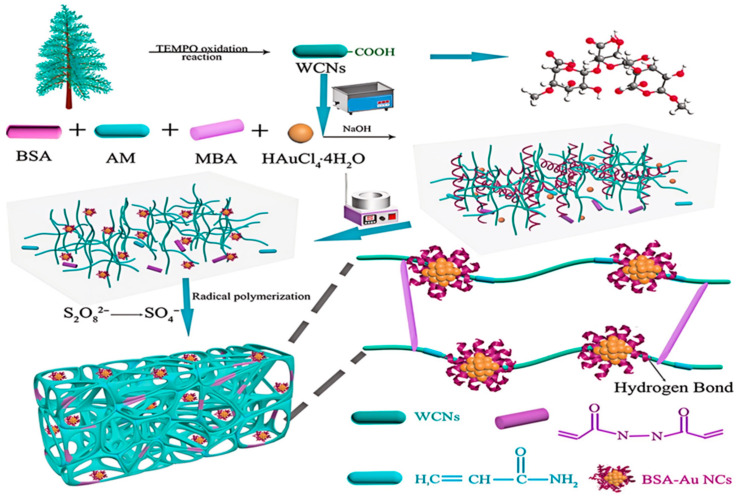
Schematic procedure for preparing a wood-derived nanocellulose hydrogel (WNH). Reprinted with permission from Ref. [51]. Copyright 2008 Elsevier.

Lei et al. have reported a fluorescent nanocellulose hydrogel (NHB) with gold nanoclusters (Au NCs) that can effectively adsorb and detect Hg(II) ions [50] (Figure 8). The NHB hydrogel had a high adsorption capacity for Hg(II), with a maximum adsorption capacity of 95.7 mg/g. It also exhibited high detection sensitivity and selectivity toward Hg(II) over the 5–150 mg/L range, with an LOD of 2.7 mg/L. The toxic Hg(II) bound to hydroxyl and carbonyl groups, concentrating on the hydrogel surface. Subsequently, they reported a wood-derived nanocellulose hydrogel (WNH) through in situ synthesized Au NCs [51] (Figure 9). The WNH composite hydrogel showed an adsorption capacity of up to 234.4 mg/g for Hg(II). It also had excellent selectivity and sensitivity toward Hg(II), accompanied by a significant fluorescence quenching, with an LOD value of 0.09 μg/L. The excellent adsorption of this WNH composite hydrogel made it ideal as a solid-state fluorescent probe for detecting Hg(II) ions. When the WNH composite hydrogel was immersed into an Hg(II)-containing solution, Hg(II) was gradually adsorbed on the interface of WNH through electrostatic interactions. The 3D porous structure in the hydrogel network facilitated Hg(II) to gradually diffuse into the interior of WNH. Simultaneously, Hg(II) accumulated within Au nanoclusters via a metal affinity effect, leading to fluorescence quenching of WNH.

**Figure 10:**
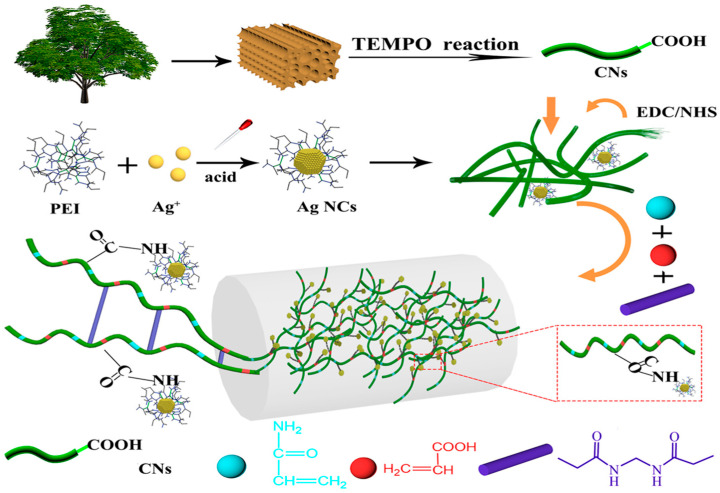
Preparation procedure of the nanocellulose composite hydrogel (NBH). Reprinted with permission from Ref. [52]. Copyright 2008 Elsevier.

Recently, another nanocellulose composite hydrogel (NBH) with silver nanoclusters was reported for the detection and removal of Cr(VI) [52] (Figure 10). The NBH hydrogel showed high adsorption for Cr(VI), with a maximum adsorption capacity of 418.5 mg/g. Its adsorption efficiency remained > 85% after four regeneration cycles. The NBH hydrogel demonstrated high selectivity and sensitivity toward Cr(VI), achieving an LOD value of 0.43 μg/L. The adsorption and detection mechanism was as follows: Cr(VI) ions were initially adsorbed onto the NBH surface due to the chelating effect. Concurrently, Cr(VI) ions were adsorbed onto the amino groups of silver nanoclusters, leading to fluorescence quenching.

As presented in Table 1, among the three CMC-based hydrogel adsorbents, the cellulose nanofiber-based hydrogels showed the most efficient adsorption of HMIs. The cellulose-based composite hydrogels showed efficient adsorption of various HMIs including Ag(I), Fe(III), Hg(II), Cr(VI) and Pb(II) with a maximum adsorption capacity of 407, 769, 846.7, 648.4 and 2056 mg/g, respectively, much larger than commercial adsorbents like activated carbon (<100 mg/g). These cellulose-based hydrogels had a high selectivity and sensitivity toward Ag(I), Fe(III), Hg(II) and Cr(VI), achieving an LOD value as low as 3.798 μM, 0.6595 mg/L, 0.09 μg/L, and 0.43 μg/L, respectively. Therefore, the cellulose-based composite hydrogels showed efficient HMI adsorption and sensitive detection. The cellulose nanofiber-based hydrogels showed more efficient HMI adsorption and sensitive detection due to the abundant heteroatom-containing functional groups on the hydrogel surface and the complex hierarchical nano-channel within the hydrogel network.

### 2.2. Chitosan-Based Hydrogels

Chitosan (CS) is a natural, abundant, biodegradable and low-cost cationic alkaline polysaccharide. It is made up of D-glucosamine and N-acetyl-D-glucosamine units with β(1→4) glycosidic bonds as a linker. CS is commonly obtained through the alkaline deacetylation of chitin, which is the second most abundant natural polymer. CS contains abundant hydroxyl and amino groups, exhibiting strong chelating activity toward metal ions [53]. This makes it an ideal biomass-based adsorbent material for water treatment and environmental detection. However, hydrogels prepared solely through the physical crosslinking of CS for use as HMI adsorbents suffer from low mechanical strength and a limited number of active sites due to their high crystallinity, poor solubility, and instability in acidic media. Various techniques have been used to tailor the characteristics of CS for adsorbing and sensing HMIs [54].

**Figure 11:**
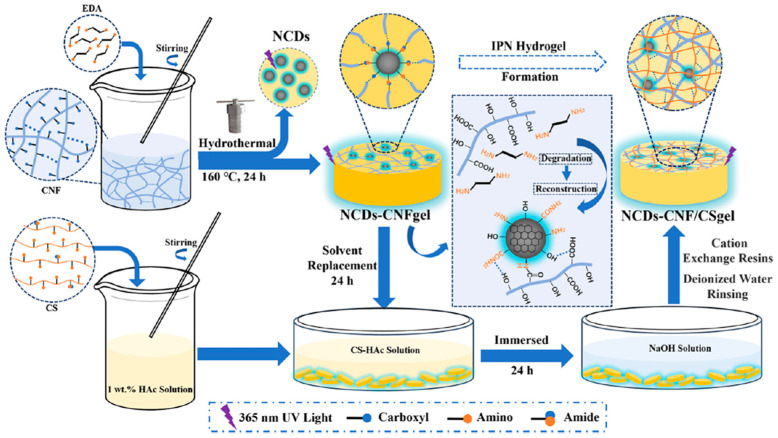
Preparation procedure and formation mechanism of the fluorescent composite hydrogel (NCDs-CNF/CS). Reprinted with permission from Ref. [55]. Copyright 2008 Elsevier.

Chen et al. prepared a composite hydrogel (NCDs-CNF/CS) with N-doped CDs (NCDs) as the crosslinking agent for adsorbing and detecting Cu(II) and Cr(VI) [55] (Figure 11). The NCDs-CNF/CS hydrogel was found to be a highly effective HMI adsorbent for Cu(II) and Cr(VI), with a maximum adsorption capacity of up to 148.30 and 294.46 mg/g, respectively. After five cycles, the adsorption capacity of NCDs-CNF/CS hydrogels remained at about 85% for Cu(II) and at about 80% for Cr(VI). The coexisting anions, cations, and organic compounds had no significant effect on the adsorption of Cu(II) and Cr(VI). The fluorescence response of the hydrogel toward Cu(II) ranged from 50 to 1000 mg/L, with an LOD value of 40.3398 mg/L. For Cr(VI), it demonstrated excellent sensitivity and selectivity, with an LOD value of 0.7093 mg/L and a linear range of 1–50 mg/L.

**Figure 12:**
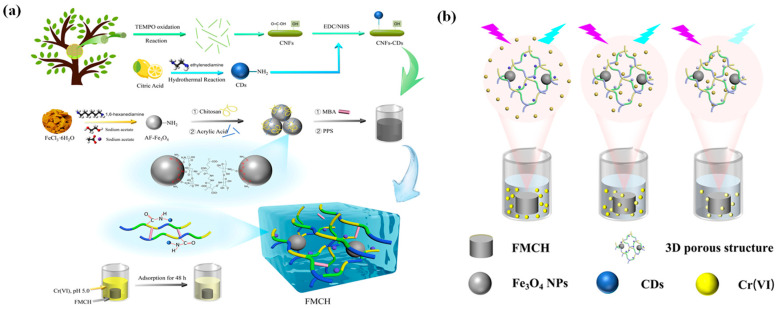
(**a**) Schematic illustration for developing a fluorescent magnetic CS-based hydrogel (FMCH). (**b**) Cr(VI) adsorption and detection mechanism of the FMCH. Reprinted with permission from Ref. [56]. Copyright 2008 Elsevier.

Luo et al. successfully synthesized a fluorescent magnetic CS-based hydrogel (FMCH) with amino-functionalized Fe_3_O_4_ nanoparticles (A-Fe_3_O_4_) and CNFs-modified-CDs for the adsorption and detection of Cr(VI) [56] (Figure 12a). The maximum adsorption capacity of the FMCH hydrogel toward Cr(VI) at pH 5.0 was 212.1 mg/g. The FMCH hydrogel exhibited a high sensitivity and selectivity toward Cr(VI), with a linear range of 20–800 mg/L and an LOD value of 14.21 mg/L. The numerous adsorption sites and ion transport channels in the FMCH hydrogel were responsible for the excellent adsorption capacity (Figure 12b). The addition of A-Fe_3_O_4_ NPs and CNFs grafted with CDs further enhanced the adsorption capacity and enabled a rapid visual response.

Wei et al. developed a family of CS-based fluorescent hydrogel films (CSBHD) with biphenyl-2,5-dicarboxaldehyde (BHD) as the crosslinking agent [57]. The CSBHD showed high sensitivity and selectivity for detecting Fe(II), with a linear range of 0–160 μM and an LOD value of 0.55 μM. Additionally, it could effectively adsorb Fe(II) with a maximum adsorption capacity of 223.5 mg/g.

Deng et al. proposed a facile and effective approach for preparing chitosan-sulfonated quantum dot (CS-SQDs) composite hydrogels for detecting and adsorbing Cr(VI) [58]. The hydrogel demonstrated good fluorescence stability under pH variations, different ionic strengths, long-time UV irradiation, and long-term storage. The CS-SQDs showed high sensitivity and selectivity for detecting Cr(VI), with an LOD value as low as 176.2 nM. Furthermore, the CS-SQDs hydrogel demonstrated a high adsorption capacity for Cr(VI) ions, achieving a maximum adsorption capacity of 186.22 mg·g^−1^.

From Table 1, Chitosan-based hydrogels showed efficient adsorption of Fe(II) and Cr(VI), with maximum adsorption capacities of 223.5 and 294.46 mg/g, respectively. These hydrogels had a high selectivity and sensitivity toward Fe(II) and Cr(VI), achieving an LOD value as low as 0.55 μM and 176.2 nM, respectively.

### 2.3. Alginate-Based Hydrogels

Alginate is a polysaccharide copolymer consisting of unbranched α-L-guluronic acid and β-D-mannuronic acid units. It is primarily extracted from the cell walls of brown algae such as kelp and giant kelp. The most common form is sodium salt, sodium alginate. Sodium alginate hydrogels are formed by the physical or chemical crosslinking of natural sodium alginate. This unique structure endows them with a series of outstanding characteristics. Alginate-based hydrogels have a high adsorption capacity and can effectively remove HMIs from wastewater [59]. The sodium alginate molecular chain is rich in carboxyl and hydroxyl groups, allowing various functional groups or polymers to be chemically grafted onto it, thereby introducing new properties.

**Figure 13:**
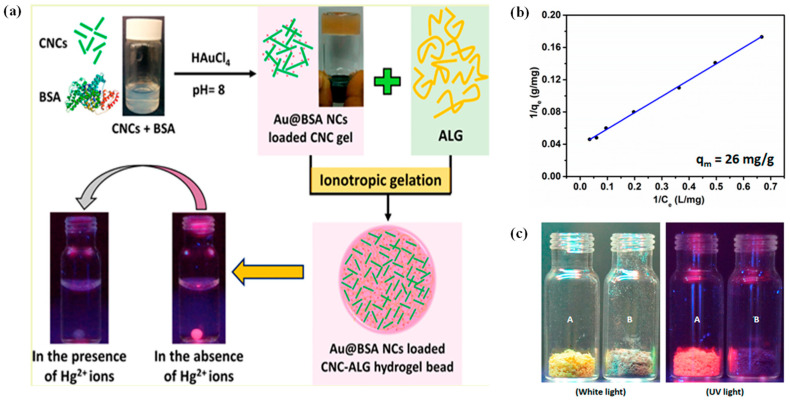
(**a**) Schematic representation for developing hydrogel beads (HB). (**b**) Equilibrium Hg^2+^ batch adsorption data, fitted using the linearized form of the Langmuir adsorption isotherm. (**c**) Images of the vials containing the nanocomposite (A) before and (B) after adsorption of Hg^2+^ ions under white light and UV light. Reprinted with permission from Ref. [60]. Copyright 2008 Elsevier.

Mohammed et al. synthesized a series of hydrogel beads (HB) capable of simultaneously sensing and removing toxic Hg(II) at low concentrations with sodium alginate, cellulose nanocrystals, and gold nanoclusters (Au@BSA NCs) as starting materials [60] (Figure 13). The hydrogel beads exhibited high sensitivity and selectivity toward Hg(II). When immersed in a 1 ppm Hg(II) solution, the fluorescence intensity of the nanocomposite was quenched due to the metallophilic Hg(II)/Au(II) interaction between Au@BSA NCs and Hg(II). Upon binding with Hg(II), the nanocomposite showed an obvious color change from yellowish-brown to dark brown. The hydrogel beads had a maximum adsorption capacity of 26 mg/g for Hg(II). The porous structure of the composite hydrogel provided numerous adsorption sites, enabling Hg(II) adsorption via electrostatic and coordination interactions. The high affinity between Hg(II) and Au(II) disrupted the fluorescence generated by Au@BSA NCs. Furthermore, Hg(II) formed Hg-S bonds with sulfur in BSA, leading to the reduction of Hg(II) to Hg and subsequent fluorescence quenching.

Zhang et al. developed a fluorescent hydrogel bead adsorbent (SA/CS_2_/N-CDs) by incorporating amino-modified carbon dots and chitosan into a CS_2_-modified sodium alginate gel. This adsorbent enabled sensitive dual-mode detection (naked-eye/fluorescence) and effective removal of Pb(II) [61]. The SA/CS_2_/N-CDs hydrogel could effectively adsorb Pb(II) with a maximum adsorption capacity of 607.08 mg/g. The fluorescence intensity of the hydrogel progressively increased with rising Pb(II) concentrations. In fluorescence detection mode, Pb(II) concentrations showed an excellent linear relationship (R^2^ = 0.993) within the range of 0–50 μmol/L, with an LOD value of 0.12 μmol/L. The embedded N-CDs on the hydrogel surface, along with abundant functional groups such as -COOH, -OH, and -SH functionalities, enhanced the aggregation and chelation of Pb(II) ions. This dual mechanism endowed the hydrogel material with both adsorption and detection capabilities.

**Figure 14:**
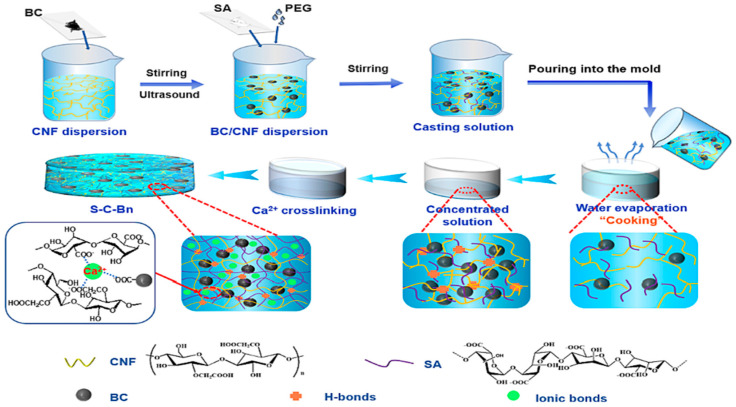
Flow chart for preparing a self-supporting, polysaccharide-based hydrogel (S-C-Bn) membrane. Reprinted with permission from Ref. [62]. Copyright 2008 Elsevier.

Cai et al. reported a self-supporting, polysaccharide-based hydrogel membrane (S-C-Bn) [62] (Figure 14). The cellulose nanofibers and micron-sized biochar were introduced into an SA hydrogel, and S-C-Bn was achieved through an in situ free water evaporation process and ionic crosslinking. The S-C-Bn hydrogel membrane achieved a flux of 61.5 L/m^2^·h under 0.35 MPa pressure during filtration testing, with removal efficiencies of 96.8% and 91.4% at 50 mg/L for Cr(III) and Cr(VI), respectively.

### 2.4. Lignin-Based Hydrogel

Lignin is the second most abundant natural organic polymer. As a key component of plant cell walls, lignin is primarily made up of three phenylpropane units (p-coumaric acid, coniferyl alcohol, and sinapic acid) linked by ether and carbon–carbon bonds [63]. Lignin’s wide availability makes it a raw material for preparing green, low-cost functional hydrogels [64]. Furthermore, its structure contains abundant functional groups such as aliphatic hydroxyls, phenolic hydroxyls, carboxyls, and quinones, which can be utilized for chemical modification and graft copolymerization. Therefore, lignin is considered a promising source of renewable material for preparing functional hydrogels. However, raw lignin tends to agglomerate in solution, hindering pollutant adsorption. It is typically chemically modified with heteroatom-containing functional groups to enhance its adsorption capacity for HMIs. Common synthetic methods for lignin-based hydrogels include physical crosslinking, solvent exchange, chemically crosslinked copolymerization and radical polymerization.

**Figure 15:**
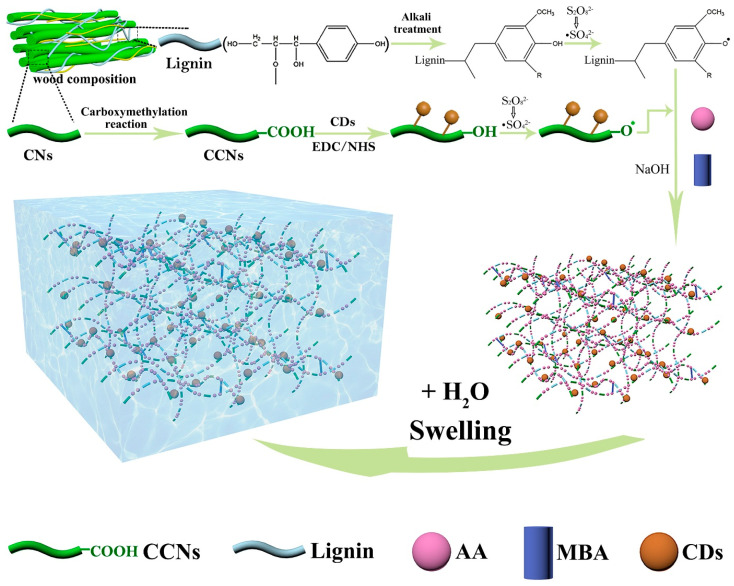
The synthesis procedure of a lignin-based hydrogel with CDs. Reprinted with permission from Ref. [65]. Copyright 2008 Elsevier.

Yuan et al. synthesized a fluorescent lignin-based hydrogel with CDs [65] (Figure 15). This hydrogel exhibited a maximum adsorption capacity for Cr(VI) of 599.9 mg/g. Concurrently, the novel hydrogel demonstrated high sensitivity toward Cr(VI), with an LOD value of 11.2 mg/L and a wide linear range of 15–200 mg/L. The 3D porous structures of the hydrogel were responsible for the adsorption and detection of Cr(VI).

**Figure 16:**
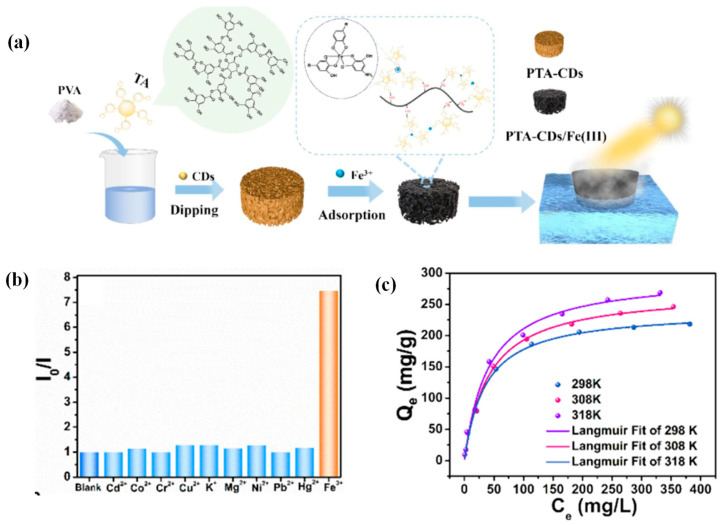
(**a**) Schematic illustration of solar steam generation. (**b**) The fluorescence ratio of the hydrogel was analyzed in the presence of various HMIs at a concentration of 200 μM. (**c**) The adsorption capacity of the hydrogel at different temperatures. Reprinted with permission from Ref. [66]. Copyright 2008 Elsevier.

Yan et al. reported a fluorescent lignin-based hydrogel for Fe(III) ion adsorption and recognition by grafting N-doped CDs onto a 3D tannic acid-based hydrogel network [66] (Figure 16). The hydrogel demonstrated high sensitivity toward Fe(III) within the range of 0–200 µM, with an LOD value of 5.4 × 10^−4^ M. The hydrogel could effectively adsorb Fe(III) ions at room temperature, with a maximum adsorption capacity of 239.2 mg/g. Notably, the saturated adsorbent material had a solar steam generation efficiency of around 2.62 kg m^−2^ h^−1^ when exposed to one sun irradiation.

**Figure 17:**
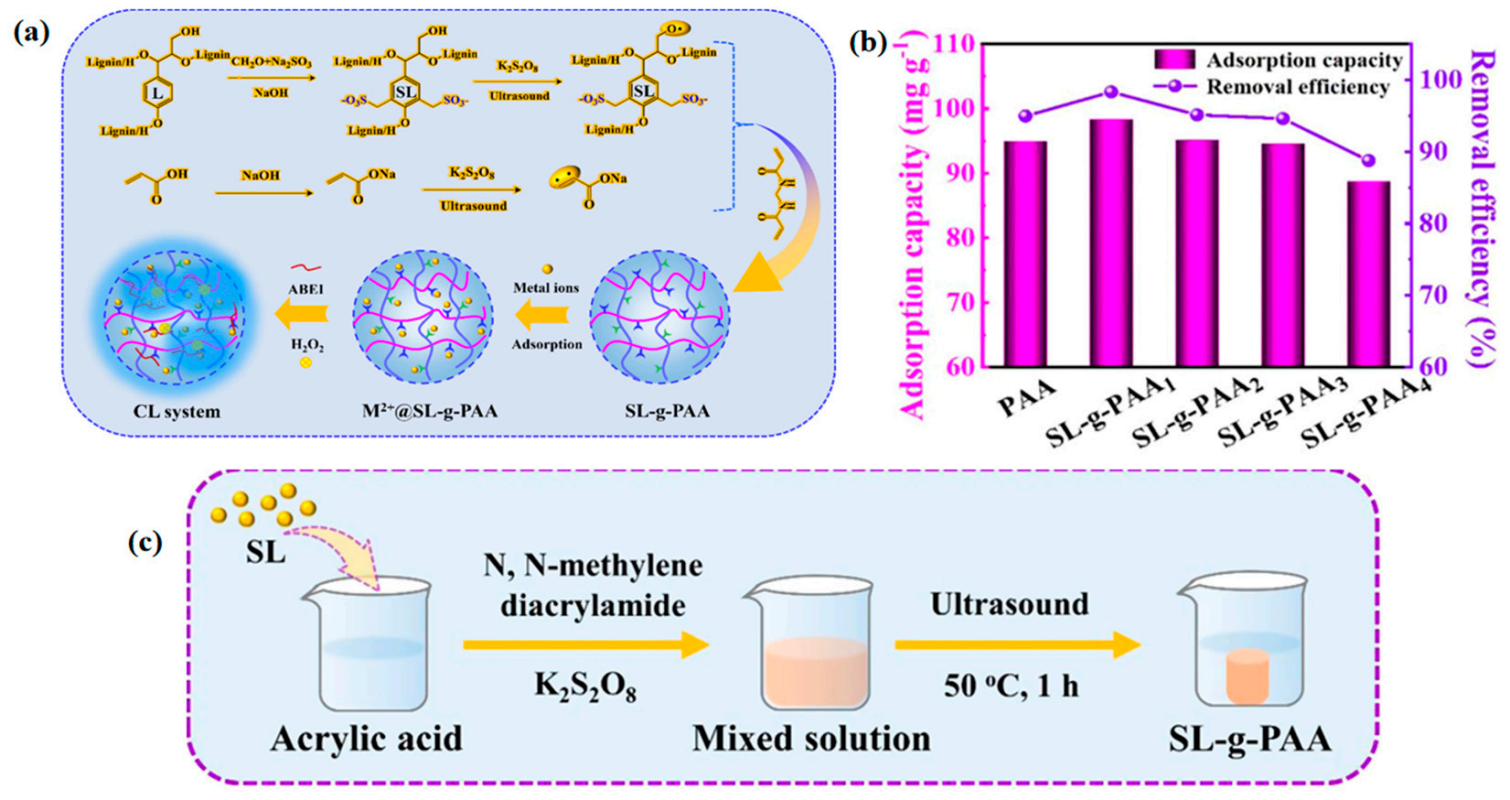
(**a**) Schematic representation for preparation and application of a lignin-based hydrogel (SL-g-PAA) for adsorption of HMIs and chemiluminescence (CL). (**b**) Adsorption capacity and removal efficiency of PAA and SL-g-PAA hydrogels for Co^2+^ ions in simulated wastewater (concentration of 200 mg Co L^−1^). (**c**) Summary of the ultrasonic-assisted synthesis of SL-g-PAA hydrogel. Reprinted with permission from Ref. [67]. Copyright 2008 Elsevier.

Jiao et al. developed a lignin-based hydrogel (SL-g-PAA) through a two-step, eco-friendly strategy [67] (Figure 17). Firstly, the lignin underwent sulfomethylation to provide more adsorption active sites and increase the polymer’s water solubility. Then, a new SL-g-PAA hydrogel was successfully produced using a free-radical polymerization process assisted by ultrasound in an aqueous environment. The prepared SL-g-PAA hydrogel showed effective adsorption of various HMIs, including Co(II), Cu(II), Ni(II), Cd(II), and Pb(II). The maximum adsorption capacities were 145.14, 117.64, 166.72, 277.89, and 344.85 mg g^−1^ for the above-mentioned toxic HMIs, respectively. Tian et al. fabricated a lignin-based hydrogel with chitosan, sodium lignosulfonate, and acrylic acid as starting materials and ammonium persulfate and N, N’-methylenebisacrylamide as the thermal initiator and crosslinking agent, respectively, by an ultrasonic-assisted method [68]. This hydrogel showed adsorption capacities of 385 mg g^−1^ and 290 mg g^−1^ for Co(II) and Cu(II), respectively, with an optimal adsorption pH value near 6.

Overall, as shown in Table 1, compared with the other three types of bio-based composite hydrogels, the cellulose-based hydrogels exhibited more efficient HMI adsorption and sensitive detection. For the toxic HMIs, including Cr(VI), Hg(II) and Pb(II), the maximum adsorption capacities were 648.4, 846.7, and 2056 mg g^−1^, respectively, much larger than those of the commercial adsorbents. The excellent HMI adsorption was attributed to the abundant heteroatom-containing functional groups on the surface of the bio-based composite hydrogel and the intricate hierarchical nano-network structure within the hydrogel. The heteroatom-containing functionalities, including -OH, -NH_2_, -COOH, and -CONH, could effectively bind with HMIs through electrostatic interactions, coordination covalent bonds, hydrogen bonding and van der Waals forces. The hierarchical nanostructure of hydrogels provides a large specific surface area conducive to heavy metal ion adsorption. Furthermore, its intricate internal network facilitates the diffusion of heavy metal ions into the hydrogel matrix. The bio-based hydrogels had a high selectivity and sensitivity toward Cr(VI), Hg(II) and Pb(II), achieving LOD values as low as 0.43 μg/L, 0.6595 mg/L, 0.09 μg/L and 0.12 μmol/L, respectively. The localized enrichment effect of heavy metal ions on the hydrogel surface, coupled with the sensitive interaction between heavy metal ions and fluorescent groups within the hydrogel, enables the hydrogel to sensitively detect heavy metal ions in wastewater.

**Table 1:** Comparison of the adsorption and detection performance of HMIs with various bio-based hydrogels.

Types of Adsorbents	Adsorbents	Types of HMI_S_	Equilibrium Adsorption (mg/g)	LOD Value	Linear Range	Reference
Cellulose-based hydrogels	BCS double network hydrogel	Ag(I)	407	3.798 μM	0–75 μM	[40]
	N, P-CDs@CMC/PEI composite hydrogel	Cr(VI)	289.5	-	-	[25]
		Hg(II)	846.7	0.48 mg/L	1–200 mg/L	
	DCG hydrogel	Fe(III)	125.30	0.6595 mg/L	0–100 mg/L	[41]
	CDs@CNFs hydrogel	Fe(III)	769	18 mg/L	-	[31]
		Pb(II)	2056	-	-	
	Nanocellulosic FNH hydrogel	Cr(VI)	648.4	0.039 μg/L	-	[44]
	CTH hydrogel	Cr(VI)	373.3 ± 14.2	-	-	[45]
	ACDs@CNFs hydrogel	Cr(VI)	534.4	4.2 mg/L	10–50 mg/L	[46]
	FNH hydrogel	Fe(III)	98.3	62.5 mg/L	-	[47]
		Pb(II)	442.0	-	-	
	CNFs-based hybrid hydrogel	Hg(II)	290.70	8.8 × 10^−8^ M	-	[48]
	NHB hydrogel with Au nanocluster	Hg(II)	95.7	2.7 mg/L	5–150 mg/L	[50]
	WNH composite hydrogel	Hg(II)	234.4	0.09 μg/L	-	[51]
	NBH composite hydrogel with silver nanoclusters	Cr(VI)	418.5	0.43 μg/L		[52]
Chitosan-based hydrogels	The NCDs-CNF/CS hydrogel	Cr(VI)	148.30	40.3398 mg/L	50–1000 mg/L	[55]
	fluorescent FMCH hydrogel	Cr(VI)	294.46	0.7093 mg/L	1–50 mg/L	[56]
		Cr(VI)	212.1	14.21 mg/L	20–800 mg/L	
	CSBHD hydrogel	Fe(II)	223.5	0.55 μM	0–160 μM	[57]
	CS-SQDs composite hydrogels	Cr(VI)	186.22	176.2 nM	-	[58]
Alginate-based hydrogels	hydrogel beads (HB)	Hg(II)	26	-	-	[60]
	SA/CS_2_/N-CDs hydrogel beads	Pb(II)	607.08	0.12 μmol/L	0–50 μmol/L	[61]
Lignin-based hydrogels	Lignin-based hydrogel with CDs	Cr(VI)	599.9	11.2 mg/L	15–200 mg/L	[65]
	fluorescent lignin-based hydrogel with N-doped CDs	Fe(III)	239.2	5.4 × 10^−4^ M	0–200 µM	[66]

## 3. Conclusions

Water pollution caused by heavy metal ions has become a worldwide issue. Hydrogels are three-dimensional networks that can retain water. Abundant, sustainable and environmentally friendly natural biomass is an ideal raw material for the preparation of hydrogels for the capture of heavy metal ions in polluted waters. Bio-based hydrogel adsorbents with ion exchange and/or chelating groups have attracted intensive interest for their ability to remove HMIs from wastewater due to their being widely available, environmentally friendly, cost effective, free from secondary pollution, biodegradable and non-toxicity. Beyond HMI adsorption, the detection of HMIs in wastewater is also crucial for environmental monitoring and human health protection. Bio-based hydrogels acted as adsorption aggregators, increasing the local concentration of HMIs and facilitating the sensitive detection of HMIs. The locally enriched HMIs could bind with the fluorescent sensor (e.g., CDs and metallic clusters) embedded in the hydrogel network even at a very low concentration, resulting in fluorescence quenching and thus achieving sensitive detection of HMIs. Therefore, bio-based fluorescent hydrogels are capable of simultaneously adsorbing and detecting HMIs. In this review, we mainly summarized the recent progress of four kinds of bio-based fluorescent hydrogels for simultaneously adsorbing and detecting HMIs: cellulose-, chitosan-, alginate- and lignin-based hydrogels. The excellent adsorption capacity was as follows: (1) the abundant functional groups such as -COOH, -OH, -NH_2_, -SO_3_H, -CONH_2_ on the hydrogel surface provide numerous adsorption active sites. (2) The 3D porous structure of the hydrogel provides a huge specific surface and various transport pathways for HMIs. (3) The insoluble nature and robust mechanical strength of the bio-based hydrogel render them easily separated from wastewater after absorbing HMIs, avoiding secondary contamination. Due to the local HMI enrichment effect, the fluorescent hydrogels can also sensitively detect HMIs in wastewater.

Although a great number of bio-based fluorescent hydrogels capable of simultaneously adsorbing and detecting heavy metal ions have been reported, many challenges remain. Firstly, some hydrogel adsorbents will lose their adsorption sites and break into pieces with increasing generation cycles. Therefore, more effort should be devoted to developing more efficient hydrogel adsorbents. Secondly, the components in the actual wastewater are relatively complex, and the presence of inorganic salts and organic acids affects the removal efficacy. The effect of various coexisting components including inorganic salts, organics, dyes, and pH values on HMI adsorption should be fully evaluated in the future. Thirdly, most reported hydrogel adsorbents were only used to adsorb one or two kinds of HMIs in deionized water in the lab. The adsorption of hydrogel adsorbents in industrial wastewater is rarely reported. Future research recommendations for bio-based hydrogel adsorbents include developing novel bio-based hydrogel adsorbents with robust mechanical strength and excellent adsorption capacity to improve their performance in wastewater treatment applications. In addition, researchers are encouraged to conduct pilot studies to validate the effectiveness of bio-based hydrogels in actual wastewater treatment processes and demonstrate their practical application at larger scales.

In summary, we provided an overview of the state of the art in bio-based fluorescent hydrogels capable of simultaneously adsorbing and detecting heavy metal ions including cellulose-, chitosan-, alginate- and lignin-based hydrogels. Given the rapid developments and increasingly sophisticated research in materials science, chemical engineering and colloid and interface science, we are confident that research into bio-based hydrogel adsorbents will continue to flourish.

## Data Availability

The data that support the findings of this study are contained within the article. More information is available on request from the corresponding author.
